# Importance and vulnerability of lakes and reservoirs supporting drinking water in China

**DOI:** 10.1016/j.fmre.2022.01.035

**Published:** 2022-03-12

**Authors:** Yunlin Zhang, Jianming Deng, Boqiang Qin, Guangwei Zhu, Yinjun Zhang, Erik Jeppesen, Yindong Tong

**Affiliations:** a State Key Laboratory of Lake Science and Environment, Nanjing Institute of Geography and Limnology, Chinese Academy of Sciences, Nanjing 210008, China; b China National Environmental Monitoring Centre, 8(B) Dayangfang Beiyuan Road, Chaoyang District, Beijing 100012, China; c Department of Ecoscience and Arctic Research Centre, Aarhus University, 8600, Silkeborg, Denmark; d Sino-Danish Centre for Education and Research, Chinese Academy of Sciences, Beijing 100101, China; e Limnology Laboratory, Department of Biological Sciences and Centre for Ecosystem Research and Implementation, Middle East Technical University, Ankara 06800, Turkey; f Institute of Marine Sciences, Middle East Technical University, Erdemli-Mersin 33731, Turkey; g School of Environmental Science and Engineering, Tianjin University, Tianjin 300072, China

**Keywords:** Centralized drinking water source, Drinking water safety, Lakes and reservoirs, Population, Water quality

## Abstract

Drinking water is closely related to human health, disease and mortality, and contaminated drinking water causes 485,000 deaths from diarrhea each year worldwide. China has been facing increasingly severe water scarcity due to both water shortages and poor water quality. Ensuring safe and clean drinking water is a great challenge and top priority, especially for China with 1.4 billion people. In China, more than 4000 centralized drinking water sources including rivers, lakes and reservoirs, and groundwater have been established to serve urban residents. However, there is little knowledge on the percentage, serving population and water quality of three centralized drinking water source types. We collected nationwide centralized drinking water sources data and serving population data covering 395 prefecture-level and county-level cities and water quality data in the two most populous provinces (Guangdong and Shandong) to examine their contribution and importance. Geographically, the drinking water source types can be classified into three clear regions exhibiting apparent differences in the respective contributions of rivers, lakes and reservoirs, and groundwater. We further found that overall, lakes and reservoirs account for 40.6% of the centralized drinking water sources *vs.* river (30.8%) and groundwater (28.6%) in China. Lakes and reservoirs are particularly important in the densely populated eastern region, where they are used as drinking water sources by 51.0% of the population (318 million). Moreover, the contribution to the drinking water supply from lakes and reservoirs is increasing due to their better water quality and many cross-regional water transfer projects. These results will be useful for the government to improve and optimize the establishment of centralized drinking water sources, which provide safe and clean drinking water in China to safeguard people's lives and health and realize sustainable development goals.

## Highlights

• Lake and reservoir contribute the most and the best centralized drinking water in China.
• Geographically, the drinking water source types can be classified into three clear regions.
• Drinking water sources will increasingly rely on the lakes and reservoirs.
• Drinking water safety will support the sustainable development of China.

## Graphical abstract

**Figure:**
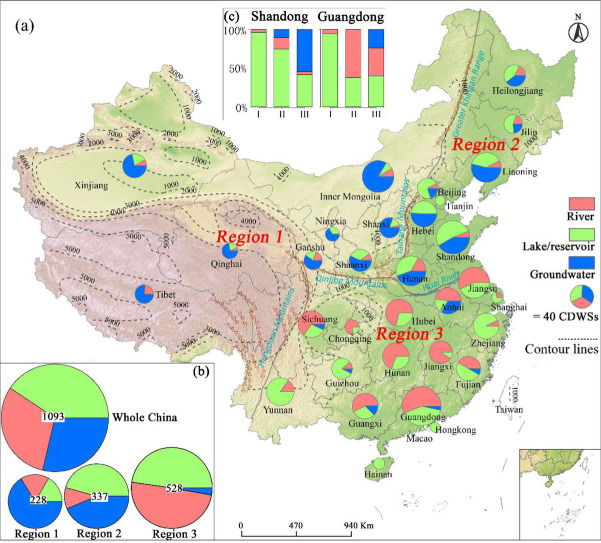

**Figure:**
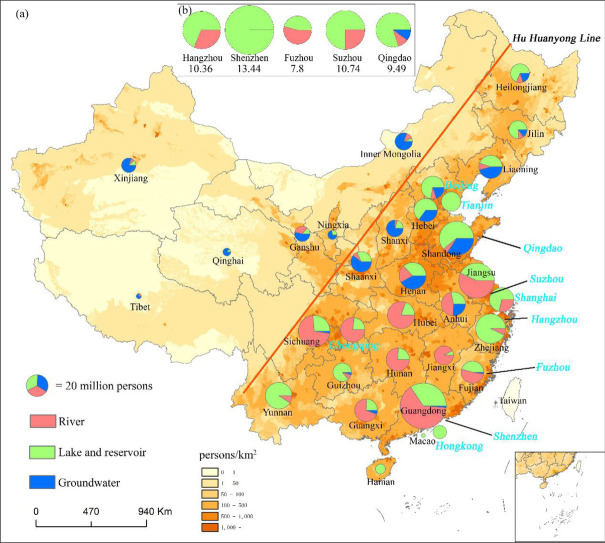

## 1 Introduction

Access to safe and clean drinking water is essential for human health, economic and social development and is recognized as the most basic requirement for sustaining livelihoods regardless of nationality, religion, color, wealth or creed [1], [2], [3]. The COVID-19 pandemic has demonstrated the critical importance of sanitation, hygiene and adequate access to clean water for preventing and containing diseases [4]. Ensuring access to clean water and sanitation for all is an important component of the United Nations 17 Sustainable Development Goals (SDGs). The first target of SDG6 is: “By 2030, achieve universal and equitable access to safe and affordable drinking water for all” [5]. It will be challenging to meet this goal because of water shortages, uneven distribution of water resources and water pollution [6]. Currently, approximately two billion people lack access to microbiologically safe drinking water [7]. Developing and least developed countries would likely suffer from additional domestic surface water deficit to achieve universal water accessibility [6]. In addition, the World Health Organization has estimated that contaminated drinking water causes 485,000 deaths from diarrhea each year (https://www.who.int/news-room/fact-sheets/detail/drinking-water). Therefore, ensuring safe and clean drinking water for communities worldwide is a great challenge [8], [9], [10], [11]. Even in countries with abundant water resources and well-developed water resource management, such as the US, drinking water pollution is considered the primary environmental concern [10]. For example, a higher level of lead was found in the blood of children who drank water from private groundwater sources, which may cause many adverse effects including intelligence quotient decrease, poor performance in school, and increased risks of behavioral problems [12]. Therefore, there are still many challenges to the continued provision of safe drinking water in the US [13].

China, a so-called “water-deficit country” that hosts 18.5% of the world's population but only 5.1% of the global freshwater suffers from a lack of clean freshwater, and 300 million people are affected by drinking water shortages [14,15]. In 2013, nearly 200 million rural residents in China did not have access to clean drinking water [16]. Poor drinking water quality affects people's lives and health [8,16,17], and every year in China, an estimated 190 million people fall ill, and 60,000 people die from diseases caused by water pollution, which costs the government $23 billion a year, or 1% of China's gross domestic product [18]. Environmental pollution, especially polluted drinking water, is considered a key factor in the occurrence of cancer villages (a phenomenon of cancer cases occurring with alarming frequency in some villages or communities) in China [19,20]. Meanwhile, water shortages and poor water quality interact with each other and further threaten China's food production and security, socioeconomic development, people's health, and realization of sustainable development goals [21]. Therefore, improving the safety of drinking water to solve these serious health and social problems is a top priority in China [17].

To reduce the problems associated with polluted drinking water, local governments have established a series of centralized and decentralized drinking water sources (CDWS and DDWS), serving cities from the prefecture to the county levels as well as towns and rural areas through pipes as a water resource management policy. A CDWS refers to a water source serving more than 1000 people by China's government, and other water sources are considered DDWSs. According to the statistics from the “National environmental protection plan for urban drinking water sources during 2008-2020” issued in 2007, China has established more than 4000 prefecture-level and county-level CDWSs and innumerable DDWSs. Consequently, 458.8 million people in urban areas and 97.9 million people in rural areas had access to improved water sources (piped water, public standpipe, protected dug well, and so on) during 1990-2015 [5]. Correspondingly, urban water use from CDWSs corresponding to domestic use and service activities has been accelerating from 1965 to 2013 [22].

There are three types of CDWSs and DDWSs in China: groundwater, rivers, and lakes and reservoirs [23]. This spatially uneven distribution of China's water resources and the inconsistency with population and local socioeconomic needs for water caused large and geographically divergent differences in CDWSs. Northern China accounts for 45.2% of the country's total population but only has 19.1% of the country's water resources [21]. Therefore, groundwater is an important drinking water resource in North China but surface water (rivers, lakes and reservoirs) is the most important drinking water resource in South Chain. Overall, the water intake from lakes and reservoirs is the largest, while that from groundwater is relatively small according to the “National Environmental Protection Plan for Urban Drinking Water Sources (2008-2020).” Although some studies have focused on the water quality and driving mechanisms of different drinking water sources [24], [25], [26], there is little knowledge on the geographical distribution, percentage, serving population and water quality of the three CDWS types. No nationwide assessment has yet to be conducted to determine the relative contribution and importance of each of these three types of CDWSs to the drinking water supply.

We collected data on CDWSs and the populations of cities at the prefecture and county levels and on the related water quality to quantify the relative contributions of three types water sources to the populations they served. Meanwhile, we included the water quality of the CDWSs in the two most populous provinces (Guangdong and Shandong) to compare the water quality and clarify the future trends of different CDWS types. Our goal is to compile the baseline knowledge on the state of CDWSs in China and highlight the vital importance of lakes and reservoirs evidenced by the CDWS number, water supply, serving population, and water quality. We expect our results to act as a foundation upon which government managers and decision-makers establish CDWSs and develop initiatives to improve drinking water quality to meet the drinking water goal of SDG6.

## 2 Data and methods

**Fig. 1:**
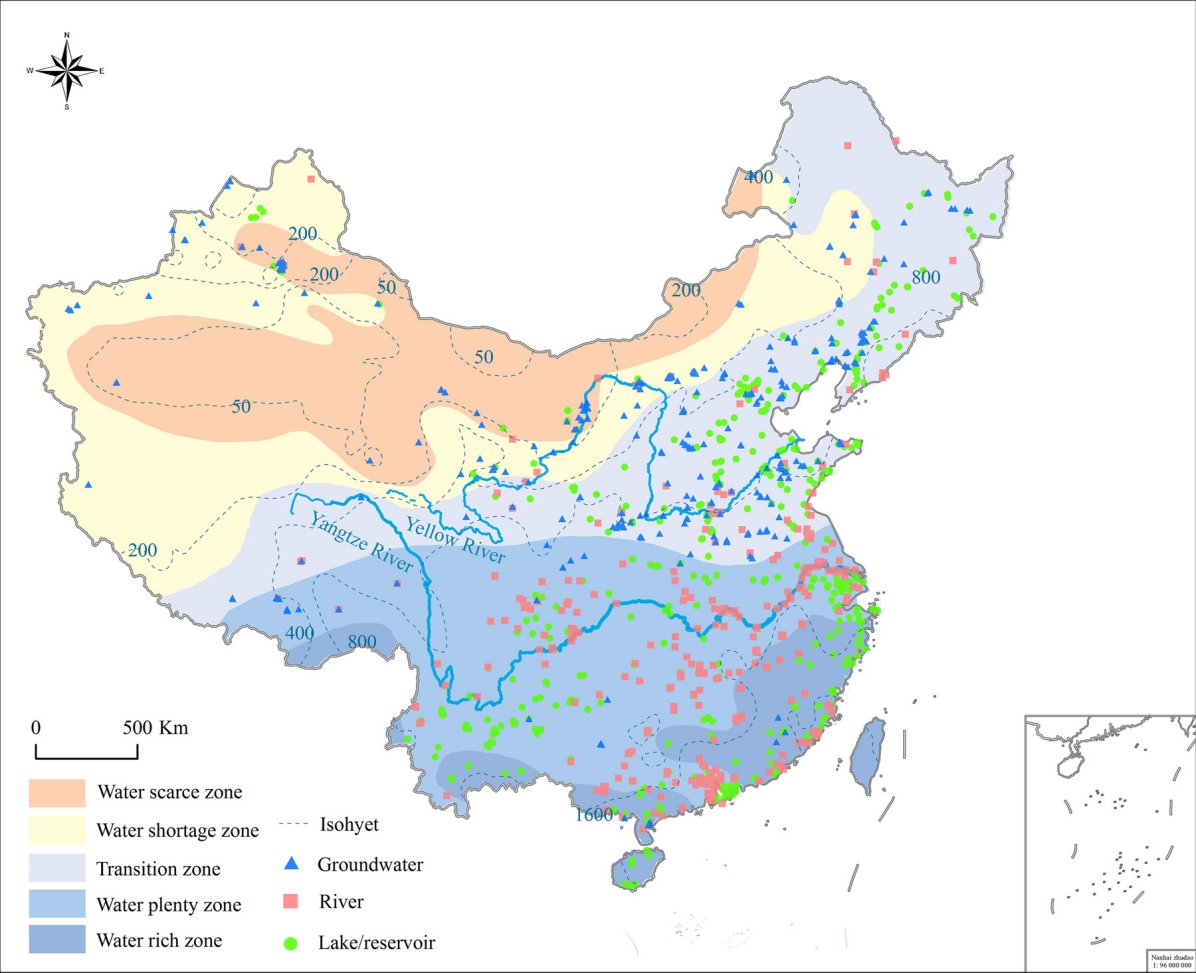
**Spatial distribution of 1093 centralized drinking water sources (lakes and reservoirs: 444; rivers: 336; groundwater: 313) collected nationwide in 340 prefecture-level and 55 county-level (population ≥1 million) cities**. Water resource region data are from the Resource and Environment Science and Data Center (https://www.resdc.cn/). Map is edited on Chinese standard map GS(2019)1823.

Our data were largely obtained from the China National Environmental Monitoring Centre and the public bulletin and reports from the Chinese government departments (Table S1), which were reliable with strict quality control. Specifically, we collected nationwide data on CDWS type and number data from 340 prefecture-level cities (including Hong Kong and Macao) and 55 county-level cities with a population greater than 1 million until 2020 (Fig. 1). We further analyzed all the county-level CDWS type, number and population data in the 10 most populous cities in China, comprising 4 municipalities directly under the Central Government (i.e. Beijing, Shanghai, Tianjin, and Chongqing), five developed and densely populated cities in 5 provinces in Southeast China (Shenzhen, Hangzhou, Fuzhou, Qingdao, and Suzhou), and Hong Kong. Additionally, we compared water quality among the three types of CDWSs (lakes and reservoirs, rivers and groundwater) from 2016 to 2020 in the two most populous provinces (Guangdong and Shandong), which have a total population of 216 million, using complete monthly records from monitoring performed by the Ministry of Ecological Environment. In China, water quality is broken into five categories that can be described as ‘‘good’’ (Grades I, II, and III) or ‘‘poor’’ (Grades IV and V or V^+^), which cannot support drinking and swimming according to the GB3838-2002 standard (Environmental Quality Standards for Surface Water).

There is no complete and available information about the CDWS type and number of all prefecture-level and county-level cities for any government department. The CDWS type and number of 338 mainland prefecture-level cities were provided by the Ministry of Ecology and Environment, China. The drinking water source type and number for Hong Kong, Macao, and the 55 county-level cities were obtained from publicly available information from local governments. The type of each water source was checked or revised manually using, for instance, new official information from the government, the latest descriptions in news reports, or telephone consultations with the person in charge of the water supply.

Population data for the prefecture-level and county-level cities in 2019 (including Hong Kong and Macao) were obtained from the Hongheiku website (https://www.hongheiku.com/), whose data are from the government statistical yearbook. The population served by each type of CDWS in the cities was calculated according to the proportion of the city's total population and the ratio of each type CDWS to total CDWS (Eq. 1). It must be clarified that the calculated population is not the actual population served by each type of CDWS because the actual population is related to the water amounts of different types of CDWSs, which is lacking in this study.

(Eq. 1) $$\mathrm{Populatio}n_{i,\;j}=\mathrm{Populatio}n_{j}\times \mathrm{CDWS}s_{i,j}\;/\;\mathrm{CDWS}s_{j}$$

where *i* is the CDWS type, such as rivers, groundwaters, lakes and reservoirs; *j* is the city name; CDWSs*_i,j_* is the number of CDWSs belonging to type *i* in city *j*; and CDWSs*_j_* is the total number of CDWSs in city *j*.

The served population of different types of CDWSs in each city, province and the whole country was calculated by summing the calculated population of each CDWS according to Eq. 1. Then the population percentage served by different CDWS types in the area is the corresponding population divided by the total population.

Monthly water quality data from 2016 to 2020 for the drinking water sources in Guangdong and Shandong provinces were obtained from the Guangdong Provincial Department of Ecology and Environment (http://gdee.gd.gov.cn/yys/index.html) and Shandong Provincial Department of Ecological Environment (http://jcc.sdein.gov.cn/hjzl/). In total, 3,791 water quality records were collected for Guangdong Province and 3,176 records were collected for Shandong Province. Surface water quality was evaluated according to environmental quality standards for surface water (GB3838-2002) with 29 indices. Groundwater quality was evaluated according to quality standards for groundwater (GB/T 14848−2017) including 39 indices. The water quality of drinking water sources is classified into five grades in China, ranging from Grade I (the best quality) to Grade V (the worst quality) according to the GB3838-2002 standard. The water quality in Guangdong and Shandong provinces generally ranged from Grade I to Grade III. Since few Grade V and Grade IV quality ratings were recorded in Guangdong and Shandong provinces, Grade IV and V were classified into Grade III in our study. Nationwide yearly water supply (surface water, groundwater, sewage treatment reuse and rainwater collection) and water use (domestic, industrial, agricultural and ecological uses) data from 1997 to 2020 were obtained from the China Water Resources Bulletin issued by the Ministry of Water Resources (http://www.mwr.gov.cn/sj/tjgb/szygb/).

To quantify the contributions of three types of prefecture-level CDWSs to the drinking water supply in Shanxi Province, monthly water intake data for 2016-2020 were obtained from the Shanxi Provincial Department of Ecological Environment (https://sthjt.shanxi.gov.cn/).

## 3 Results

### 3.1 Contributions and spatial distributions of different drinking water sources

**Fig. 2:**
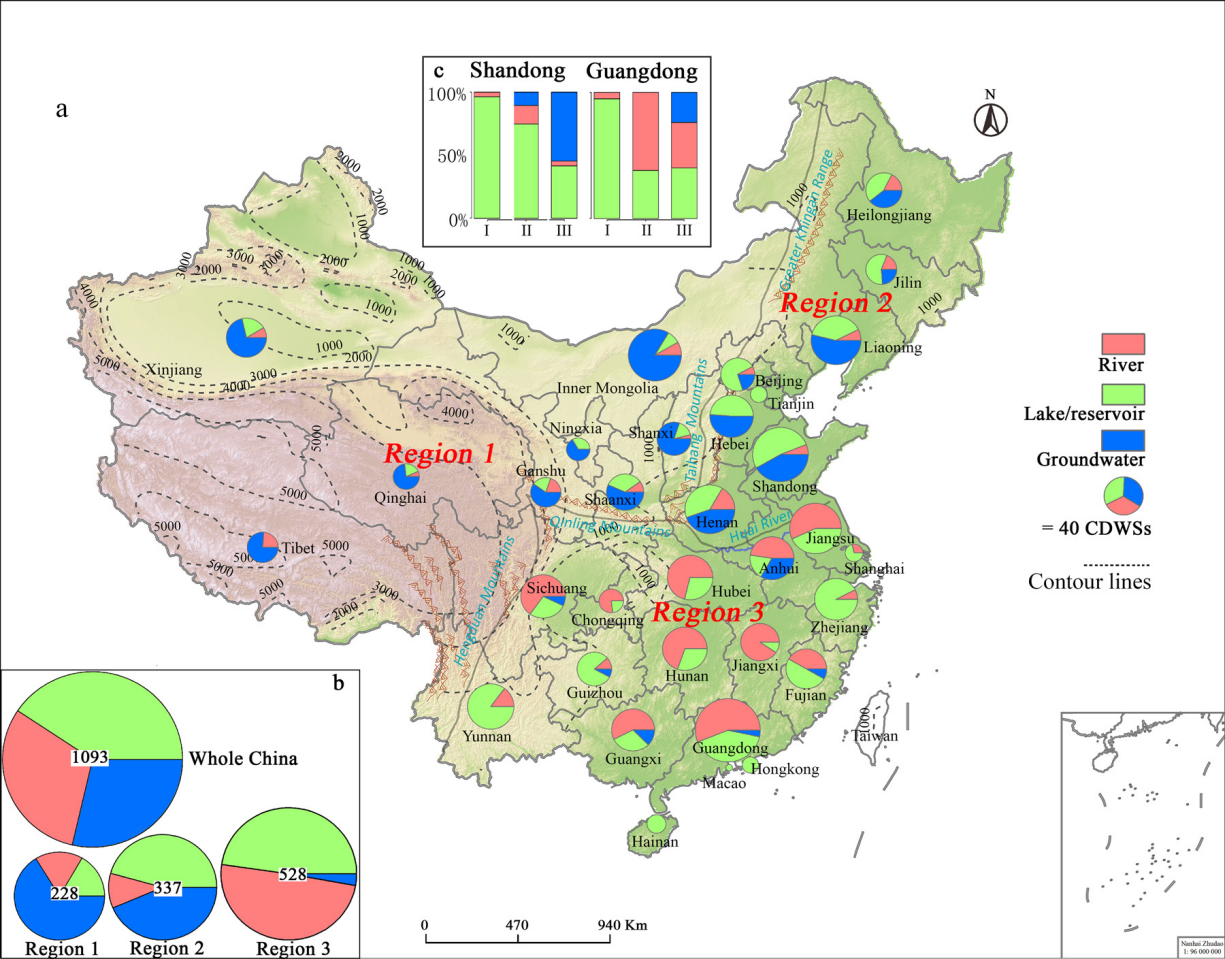
**Spatial distribution and composition of three types of drinking water sources across China (a) and within China and three geographically distinct regions (b)**. The number in the circles in (b) indicates the total number of drinking water sources in that region. Multiyear (2016-2020) average percentages of drinking water quality were calculated for Shandong and Guangdong provinces (c). Map is edited on Chinese standard map GS(2019)1823.

Of the 1,093 CDWSs nationwide (Fig. 1), 444 (40.6%) were lakes and reservoirs, 336 (30.8%) were rivers, and the remaining 313 (28.6%) were groundwater sources (Fig. 2). In addition, a more detailed analysis of the 10 most populous cities in China, including 211 county-level CDWSs, of which 138 (65.4%) were lakes and reservoirs, 65 (30.8%) were rivers, and 8 (3.8%) were groundwater, indicated the dominant role and importance of lakes and reservoirs (Table 1).

**Table 1:** **Amount, percentage, and serving population percentage of three CDWS types of the 10 important cities in China**.

Name	CDWS number	Number of three CDWS types			Serving population percentage of three CDWS types (%)		
		Lakes and Reservoirs	Rivers	Groundwater	Lakes and Reservoirs	Rivers	Groundwater
Beijing	25	18	2	5	72	8	20
Shanghai	7	5	2	0	71.4	28.6	0
Tianjin	6	6	0	0	100	0	0
Chongqing	73	36	37	0	49.3	50.7	0
Shenzhen	12	12	0	0	100	0	0
Hangzhou	16	11	5	0	68.8	31.2	0
Fuzhou	24	11	13	0	45.8	54.2	0
Qingdao	30	24	3	3	80	10	10
Suzhou	12	9	3	0	75	25	0
Hong Kong	6	6	0	0	100	0	0
All	211	138	65	8	72.9	23.6	3.5

**Fig. 3:**
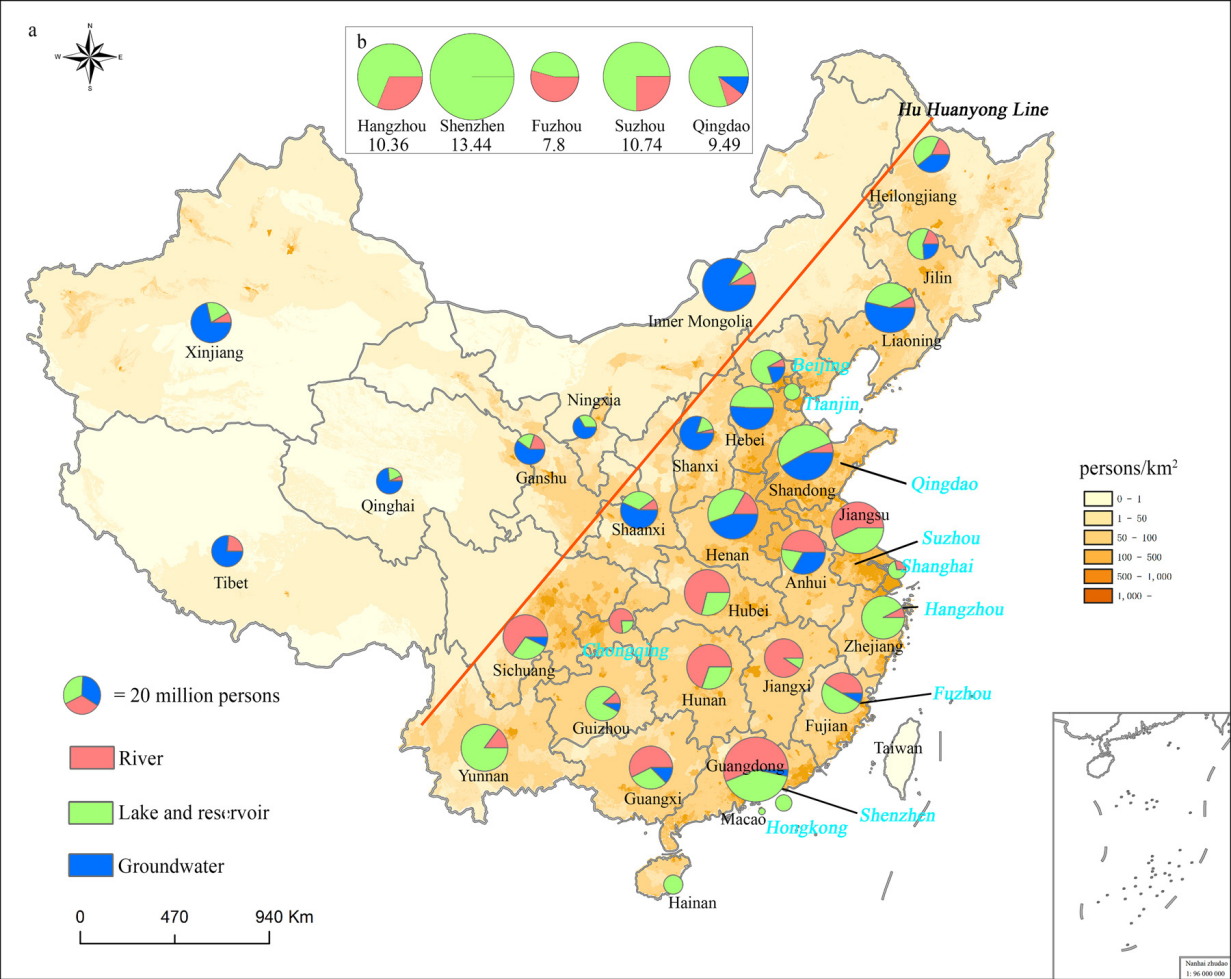
**Distribution of the population relative to the CDWSs available in the provinces, four municipalities and large cities (Beijing, Shanghai, Tianjin, Chongqing, and Hong Kong)** (a) as well as in five other populous cities in Southeast China. The number below each city indicates its population (in millions of people) (b). Map is edited on Chinese standard map GS(2019)1823.

Geographically, the CDWS types could be clearly classified into three regions from the perspectives of topography and water resource distribution (Fig. 1), separated by China's Second Ladder and the Qinling Mountains-Huaihe River Line (Fig. 2a). In the first region located in water scarce and shortage regions, groundwater is the main source of drinking water (66.2%); this region coincides approximately with the sparsely populated western region of China (Figs. 2b and 3). For example, the groundwater water source accounted for 80.8% of the total CDWSs in Shanxi Province (Table 2). The altitude of the first region is generally above 2000 m, and the annual average rainfall is generally less than 400 mm. Due to high evaporation and low precipitation, most of the lakes in this area are saltwater lakes or brackish water lakes. Meanwhile, many rivers are cut off and frozen in winter due to low discharge and temperature. All these factors inhibited lakes and rivers as CDWSs. In the second region located in the transition region from water resource shortages to plenty, in the moderately populated northeast, both lakes and reservoirs (45.7%) and groundwater (43.6%) are important drinking water sources (Fig. 2b). The altitude of the second region is basically 1000-2000 m, and the annual average rainfall is 400-1000 mm. Many rivers are also frozen in winter and the water supply is unstable over time in this region inhibiting rivers as CDWSs. In the third region located in regions with abundant water resources and rich regions, in the densely populated south, both rivers (49.4%) and lakes and reservoirs (47.7%) are the two main drinking water sources (Fig. 2b). The altitude of the third region is generally under 1000 m, and the annual average rainfall is greater than 800 mm. Abundant freshwater resources with complex river and lake networks facilitated rivers, lakes and reservoirs as CDWSs. In addition, the drainage basins in southern China such as Fujian, Zhejiang and Guangdong provinces are usually small in area and short in flow. Reservoirs have been widely built to store water before flowing into the sea. Thus, our results revealed large and geographically divergent trends for Chinese drinking water sources.

**Table 2:** **Comparison of the percentages of CDWS number, annual total water intake amounts and water quality compliance rates of three CDWS types in Shanxi Province during 2016-2020**.

	Year		Rivers	Lakes and reservoirs	Groundwater	Total
CDWS number			1	4	21	26
Percentage of three CDWS types (%)			3.8	15.4	80.8	100
Water quality compliance rate (%)			0	100	95.2	92.3
Annual total water intake amount (10^4^ m^3^)		2016	2233	12729	29158	44120
		2017	2525	12903	30549	45977
		2018	2160	13682	32694	48536
		2019	2160	13337	33598	49095
		2020	2160	14557	33790	50506
Water intake amount from each CDWS (%)		2016	5.1	28.8	66.1	100
		2017	5.5	28.1	66.4	100
		2018	4.4	28.2	67.4	100
		2019	4.4	27.2	68.4	100
		2020	4.3	28.8	66.9	100

Water quality compliance rate: The percentage of water quality is up to or better than the Grade III standard according to the GB3838-2002 standard.

### 3.2 Water quality comparison of different drinking water sources

Our results showed that the water quality of lake and reservoir CDWS was better than that of river and groundwater CDWSs for two most populous provinces (Guangdong and Shandong Provinces). Of the Grade I (best quality) CDWSs, lakes and reservoirs constituted >90% and rivers constituted <10%; however, no groundwater sources achieved this grade (Fig. 2c). In detail, 75% of the CDWSs classified as Grade II in Shandong Province were lakes and reservoirs. In Guangdong Province, rivers accounted for the majority of the Grade II CDWSs, followed by lakes and reservoirs. In Shandong, the Grade III CDWSs were mainly groundwater, followed by lakes and reservoirs (Fig. 2c), while in Guangdong, the proportions of the three CDWS types were roughly equal for Grade III (Fig. 2c).

### 3.3 Serving population of different drinking water sources

The serving population results of the three types of CDWSs further highlight the vital importance of lakes and reservoirs for the drinking water supply. In 2019, there were approximately 701 million inhabitants in 340 prefecture-level and 55 county-level cities. Of these inhabitants, nearly 331 million (47.2%), 258 million (36.8%), and 112 million (16.0%) relied on lakes and reservoirs, rivers, and groundwater, respectively, as their drinking water sources (Fig. 3). To further underline the importance of lakes and reservoirs, special attention was given to the difference between the southeastern and northwestern regions of China, as divided by the Hu Huanyong Line [27] (Fig. 3). This line, proposed by the famous population geographer Hu Huanyong, connects the city of Aihui County (Heilongjiang Province) in the northeast to Tengchong County (Yunnan Province) in the southwest, basically coinciding with the 400-mm precipitation isoline. The Hu Huanyong Line is regarded as an important divider of geographic, economic, social, and human activity intensity in China [28]. To the southeast of the Hu Huanyong Line, 318 million (51.0%) people rely on lake and reservoir water sources, while 235 million (37.8%) and 70 million (11.0%) people are served by river and groundwater water sources, respectively (Fig. 3), which indicates the predominant surface water source. In contrast, to the northwest of the Hu Huanyong Line, 13 million (16.8%), 23 million (29.2%), and 42 million (54.0%) people are served by lake and reservoir, river and groundwater water sources, respectively. In addition, in the 10 most populous cities, 111 million inhabitants (72.9%) use lakes and reservoirs as drinking water sources, whereas only 36 million (23.6%) and 5.3 million (3.5%) rely on rivers and groundwater, respectively, as drinking water sources (Fig. 3) (Table 1).

## 4 Discussion

### 4.1 Advantages of lake and reservoir CDWSs

Drinking water quality is affected by the quality of source water, the treatment processes of waterworks, and the water distribution system. Undoubtedly, the quality of source water plays a critical role in determining drinking water quality and residents' health. Our CDWS number, water quality and serving population results all show that lakes and reservoirs determine the drinking water supply and serve the largest urban population in China. Lakes and reservoirs act as important drinking water sources around the world. For example, the Great Lakes provide drinking water to over 24 million US and Canadian consumers [29] and the Catskill/Delaware reservoirs supply drinking water for 90% of the population of New York City [30]. Drinking water security has been a major concern in China in recent decades [3,11,17]. With the increases in the population and per capita water consumption, the amount of domestic water use underwent a yearly mean increase of 2.68%, increasing the domestic water use percentage from 9.4% to 14.8% during 1997-2020 (Table S2). Therefore, ensuring a safe and clean drinking water supply remains a high priority for the Chinese government. China has 16,719 lakes and 14,001 reservoirs (water area ≥ 0.1 km^2^) [31] and most are located in the developed areas in eastern China (Figs. 2 and 3), which can provide the most important and the best quality drinking water. We will highlight the importance of lake and reservoir CDWSs in the clean and safe drinking water supplies from the three perspectives of water supply, water quality and future trends.

First, lakes and reservoirs contribute the most CDWSs (40.6%) and serve the largest population (47.2%) in China. The China Water Resources Bulletin showed that the relative contribution of groundwater to the total water supply decreased from a maximum of 19.7% in 2001 to a minimum of 15.4% in 2020 during the period from 1997-2020 (Table S2). In contrast, the relative contribution of rivers, lakes and reservoirs to the total water supply increased markedly in this period (Table S2). In Jiangsu Province, 20 groundwater sources and 18 river sources were removed from the county-level CDWSs in 2020 because the water quality did not meet national standards; additionally, 4 lakes and reservoirs were added due to the cleaner and safer drinking water supply (http://www.jiangsu.gov.cn/art/2020/9/30/art_64752_9528158.html?gqnahi=affiy2). At present, there are no county-level groundwater CDWSs in Jiangsu Province.

In addition, the water intake amount and percentage from groundwater are very low although the percentage of groundwater CDWS number is very high for some northwestern provinces. We further chose the most populous province (Shanxi Province) from 6 provinces with groundwater CDWS percentages greater than 60%, including Inner Mongolia, Shanxi, Tibet, Qinghai, Xinjiang, and Ningxia Provinces, to quantify the contribution of the water intake amounts associated with different CDWSs. A total of 80.8% of the groundwater CDWS number corresponded to 66.1%-68.4% of the total water intake amount from groundwater during 2016-2020 (Table 2). In contrast, lakes and reservoirs accounted for 15.4% of the CDWSs and 27.2%-28.8% of the total water intake amount in Shanxi Province (Table 2), indicating an underestimation of the serving population of lakes and reservoirs. The yearly mean increase rate of 2.89% for the annual total water intake amount during 2016-2020 in Shanxi Province showed an increase in the domestic water use amount (Tables 2 and S2), which indicated more water intake from lakes and reservoirs. In Huhhot, the capital of Inner Mongolia, there are 9 groundwater CDWSs and 1 river CDWS, which serve only 2.18 million people. These results indicate that the water intake amount and the percentage of the population served by groundwater CDWSs are much lower than anticipated for these northwestern provinces because of the limited water intake of a single groundwater CDWS.

Second, lakes and reservoirs provide better drinking water quality and stable water supply than rivers and groundwater (Fig. 2c and Table 2). Improved water quality is vital for eliminating disease and ensuring physical health [11,17,19,20]. Many lakes and reservoirs are far from cities and industrial areas, are free from industrial, agricultural and domestic pollution and have relatively good water quality. Previous surveys reported that nationwide, 17.1% of the groundwater but only 4.4% of the surface water CDWSs failed to meet national quality standards (GB 3838, GB/T 14848) in 2017, emphasizing the higher quality of surface waters. As much as 90% of China's shallow groundwater is polluted, according to the Ministry of Land and Resources, and an alarming 37% is so polluted that it cannot be treated for use as drinking water [18]. Similarly, China's Ecological Environment Condition Report of 2020 (http://www.mee.gov.cn/hjzl/sthjzk/) showed that 86.4% of the nation's groundwater (10171 wells in 31 provinces across China) was poor or very poor quality (i.e., class Ⅳ or V), which was not safe for drinking water. However, only 16.6% of the nation's surface water (1937 stations across China) was poor or very poor quality (i.e., class Ⅳ or V). More importantly, China is a hotspot for arsenic, and it is estimated that 4.3-19.6 million people are affected by arsenic-contaminated groundwater [32,33]. For the surface water CDWSs, water quality is better for lakes and reservoirs than rivers (Fig. 2c) [23]. Due to their better water quality, lakes and reservoirs have become the main water sources for bottled water, which now represents the fastest growing form of safe and clean drinking water in China [7,34]. For example, as the largest brand of bottled water in China, NongFu Spring has eight high-quality water sources nationwide, including Lake Qiandaohu, Lake Wanlvhu, and Danjiangkou Reservoir and the other five water sources come from high mountain springs and ice melt water without river or groundwater sources.

Additionally, lakes and reservoirs can provide more stable water supply (with fewer fluctuations due to floods, droughts and freezing) than groundwater and rivers because many groundwater bodies in China have been overextracted and polluted by a high geological levels of arsenic and/or significant human activities [18,33,35], and rivers are vulnerable to pollution [36] (Table S3), floods, droughts and freezing [37], [38], [39]. There were 653 representative cases of surface water pollution accidents before 2012, of which 567 occurred in rivers, 30 occurred in lakes and 56 occurred in reservoirs [36], indicating a greater environmental risk for river CDWSs. To the best of our knowledge, most of the major drinking water pollution incidents in China in the past 20 years have occurred in rivers (Table S3). The Songhua River industrial disaster on the upper reach of the Songhua River, the largest tributary of the Heilong River, is a typical example of an accident in a river that polluted river CDWSs. Authorities shut down the drinking water system serving Harbin's 4 million residents as well as municipal and residential groundwater supplies along the river's edge for 4 days because an explosion at a chemical plant led to the release of more than 100,000 kg of benzene, aniline, and nitrobenzene into the Songhua River [17]. Although marked water quality improvement has been achieved for China's rivers in the past two decades, it is still challenging to realize acceptable ecological conditions [40]. Meanwhile, it has been observed and projected that there are more frequent and intense floods and droughts in China [41,42], which will inevitably cause negative effects on water quality, increase vulnerability, and limit clean and safe drinking water for river CDWSs [43], [44], [45].

Third, many cross-regional water transfer projects are increasing the contribution and importance of lakes and reservoirs to CDWSs. China has been facing increasingly severe water scarcity, especially in North China due to the uneven distribution of water resources [21]. Therefore, many cross-regional water transfer projects have been implemented to ensure water resource security and further improve food security, economic development, and people's health, although such transfers may have negative impacts on local ecosystems and water resources. The Middle Route Project of the South-to-North Water Diversion (SNWD) Project, which transports water from the Danjiangkou Reservoir (connected to the Yangtze River) to Henan and Hebei Provinces and to the Tianjin and Beijing municipalities due to serious water scarcity in these regions [21] (Fig. 1), further underlines the role of lakes and reservoirs in providing drinking water in China. Since 2014, the drinking water sources in the two provinces and two municipalities have shifted from mainly groundwater to lakes and reservoirs, e.g., the Danjiangkou Reservoir [35]. In 2019, a total of 6.22 km^3^ of water was transported from Danjiangkou Reservoir to meet the demand for safe and clean drinking water, and another 2.40 km^3^ of water was transported for ecological use to restore groundwater levels for Henan and Hebei Provinces and to the Tianjin and Beijing municipalities [35]. Specifically, of this transported water, 1.22 km^3^ of drinking water was transported to Tianjin municipality in 2019, affecting 14 administrative districts. More importantly, the overall water quality in the Danjiangkou Reservoir is good always maintaining grades I and II according to the GB3838-2002 standard [46]. Additionally, a total of 34.053 km^3^ of water has been transported along the Middle Route Project of the SNWD since 2014 (http://www.mwr.gov.cn/xw/sjzs/202011/t20201103_1461936.html) (Fig. S1 and Table S4). In the future, even more water will be transported from the Danjiangkou Reservoir, Lake Hongzehu, and Lake Luomahu to the North China Plain along the East and Middle Route Projects of the SNWD. For example, the Middle Route Project of the SNWD is projected to transport 9.5 km^3^/year, with the long-term goal of transporting 13 km^3^/year, which is expected to be realized in 2030 [35]. Moreover, some major water transfer projects, such as the Lake Qiandao Water Distribution Project, the water diversion project from the Hanjiang River to the Weihe River, and the water diversion project from the Datong River to the Huangshui River, will transport water from lakes and reservoirs to ensure compliance with the safety standards for drinking water for many large and medium-sized cities (Table S5). It is projected that 17.14 km^3^/year of water will be transported from the eight cross-regional water transfer projects until 2025 benefiting more than 173.6 million people (Table S5). Meanwhile, the Anhui provincial government is implementing the water diversion project in northern Anhui to replace underground sources using rivers, lakes and reservoirs for 30 million people of Fuyang, Huaibei, and Bozhou cities before 2025. Therefore, we predict that in the future, drinking water sources will increasingly rely on the lakes and reservoirs associated with national water network backbone projects and cross-regional water transfer projects showing the increasing importance of lakes and reservoirs.

### 4.2 Risks of lake and reservoir CDWSs

Lakes and reservoirs also face risks of harmful algal blooms and water quality deterioration due to intense human activities and climate warming [47], [48], [49], [50], [51], which might increase the vulnerability of lakes and reservoirs supporting drinking water. For example, a drinking water source contaminated by harmful cyanobacteria blooms in Lake Taihu resulted in the interruption of the drinking water supply of two million people in Wuxi in Jiangsu Province for one week (Table S3) [3,52]. Additionally, similar drinking water intake contamination by harmful cyanobacteria blooms was reported in the municipality of Toledo, Ohio (US), in early August 2014, which directly affected over 400,000 residential customers and hundreds of businesses [53,54]. Freshwater lakes and reservoirs are at risk of long-term salinization due to increasing urbanization and associated chloride runoff, drought, and agricultural irrigation [55], [56], [57], [58], which will threaten drinking water supply. Preventing salinization of freshwater lakes and reservoirs is critically important for protecting the ecosystem services of freshwater lakes and reservoirs such as drinking water, fisheries, recreation, irrigation, and biodiversity maintenance. In addition, restoration of polluted lakes to improve water quality and ecological conditions needs a longer time and more effort than polluted rivers due to low hydraulic flushing rates and accumulated internal pollution from sediment [59], which pose a potential risk for lake and reservoir CDWSs. However, the vulnerability can be largely decreased with strict water source conservation and efficient water use. Especially, the Chinese government has made great efforts to control lake and reservoir eutrophication and algal bloom over the past few decades.

### 4.3 Drinking water quality management for sustainable development

Our results clearly show that lakes and reservoirs act as drinking water sources for more inhabitants than rivers and groundwater, especially in the densely populated and economically developed region of eastern China. These results were different from the previous prevailing understanding that rivers and groundwater were the most important sources of drinking water in China during 2005-2009 [23]. The percentage of lake and reservoir sources increased from 27.2% to 40.6% but the percentage of groundwater sources decreased from 42.2% to 28.6% from 2005-2009 to 2020 [23]. With China's rapid urbanization and an increase in its water use [22], it is projected that the contribution of lakes and reservoirs to drinking water sources will further increase as previously shown. The proportion of the population using piped water supplies increased from 88% to 90% in urban areas, and from 26% to 62% in rural areas between 2000 and 2015. However, 10% and 38% of the population in urban and rural areas still uses a nonpiped water supply [60]. Our dataset and results are vital for a better understanding of the roles of the different CDWSs and can be used to support the government and the public in addressing the increasing challenges affecting drinking water security in China. Our results further highlight the important ecological services and goods providing safe and clean drinking water by lakes and reservoirs for human welfare [56,61,62].

Therefore, we expect that our results will encourage the government to undertake adaptive management to ensure drinking water safety and achieve SDG6. Specifically, water conservation, protecting surface and groundwater resources, and preventing overexploitation are the priorities for addressing water scarcity and shortage in Regions 1 and 2. Meanwhile, cross-regional water transfer projects are considered an important adaptive management policy to increase the water supply and meet the increasing need. For Region 3, special attention to controlling water pollution and lake eutrophication to reduce the adverse effects of human activities on drinking water sources should be priorities. Cleaning drinking water sources, e.g., by purging rivers and lakes of industrial and agricultural pollutants, is considered a sustainable plan for maintaining China's drinking water supply [63]. Bloom prevention and control and water quality management strategies should be developed to protect lake and reservoir drinking water sources, and these strategies should include nutrient management, hydrodynamic changes, and chemical and biological control [64]. In particular, agricultural applications of pesticides and fertilizers should be limited, and polluting industries should not be allowed near important lake and reservoir drinking water sources to maintain excellent water quality.

## 5 Conclusion

The centralized drinking water source types exhibit significant spatial heterogeneity and can be classified into three clear regions across China. Lakes and reservoirs are the most and the best centralized drinking water sources contributing 40.6% of the CDWSs *vs.* river (30.8%) and groundwater (28.6%) in China, especially in the eastern developed areas serving 51.0% of the population. In the 10 most populous cities, 111 million inhabitants (72.9% population) rely on lakes and reservoirs as drinking water sources. The water quality of lakes and reservoirs is better than that of rivers and groundwater. Meanwhile, groundwater has been overextracted and rivers are vulnerable to pollution, floods, and droughts. Therefore, lakes and reservoirs can provide more stable and clean drinking water. We predict that centralized drinking water sources will rely more on lakes and reservoirs in the future accompanying the construction and operation of some cross-regional water transfer projects. Under the dual stress of climate change and human activities, lakes and reservoirs are facing the vulnerability issuses of water resources supply. Therefore, preventing lake and reservoir pollution and improving the water quality of lake and reservoir are critically important for achieving SDG6 and protecting people's lives and health.

## Author contributions

B.Q.Q designed the study. Y.L.Z., J.M.D., G.W.Z., and Y.J.Z. collected and analyzed the samples. Y.L.Z. and J.M.D. interpreted the data. Y.L.Z., J.M.D., B.Q.Q., and E.J. plotted the figures and wrote the manuscript.

## Data availability

Most data generated or analysed during this study are included in this published article and the Supplementary Information. The dataset of specific name, geographical location and type of every CDWS will be provided upon reasonable request.

Spatial distribution and composition of three drinking water source types across China.

Distribution of the population relative to the CDWSs available in the provinces, four municipalities and large cities (Beijing, Shanghai, Tianjin, Chongqing, and Hong Kong) (a) as well as in five other populous cities in Southeast China. The number below each city indicates its population (in millions of people) (b).

## Declaration of competing interest

The authors declare that they have no conflicts of interest in this work.

## Appendix Supplementary materials

Image, application 1
