# Supplementary material for: Importance and vulnerability of lakes and reservoirs supporting drinking water in China
Source: Fundam Res. 2022 Mar 12;3(2):265–73. doi: 10.1016/j.fmre.2022.01.035 (PMC11197511; doi:10.1016/j.fmre.2022.01.035)
Supplement: Supplementary file 1 [file mmc1.docx]

**Supporting materials**

**Importance and vulnerability of lakes and reservoirs supporting drinking water in China**

Yunlin Zhang^a1*^, Jianming Deng^a1^, Boqiang Qin^a^, Guangwei Zhu^a^, Yinjun Zhang^b^, Erik Jeppesen^c, d, e, f^, Yindong Tong^g^

^a^ *State Key Laboratory of Lake Science and Environment, Nanjing Institute of Geography and Limnology, Chinese Academy of Sciences, Nanjing 210008, China*

^b^ *China National Environmental Monitoring Centre, 8(B) Dayangfang Beiyuan Road, Chaoyang District, Beijing 100012, China*

^c^ *Department of Ecoscience and Arctic Research Centre, Aarhus University, 8600, Silkeborg, Denmark*

^d^ *Sino-Danish Centre for Education and Research, Chinese Academy of Sciences, Beijing 100101, China*

^e^ *Limnology Laboratory, Department of Biological Sciences and Centre for Ecosystem Research and Implementation, Middle East Technical University, Ankara 06800, Turkey*

^f^ *Institute of Marine Sciences, Middle East Technical University, Erdemli-Mersin 33731, Turkey*

^g^ *School of Environmental Science and Engineering, Tianjin University, Tianjin 300072, China*

^∗^ Corresponding author.

E-mail address: ylzhang@niglas.ac.cn (Y. Zhang).

^1^ These authors contributed equally to this work.


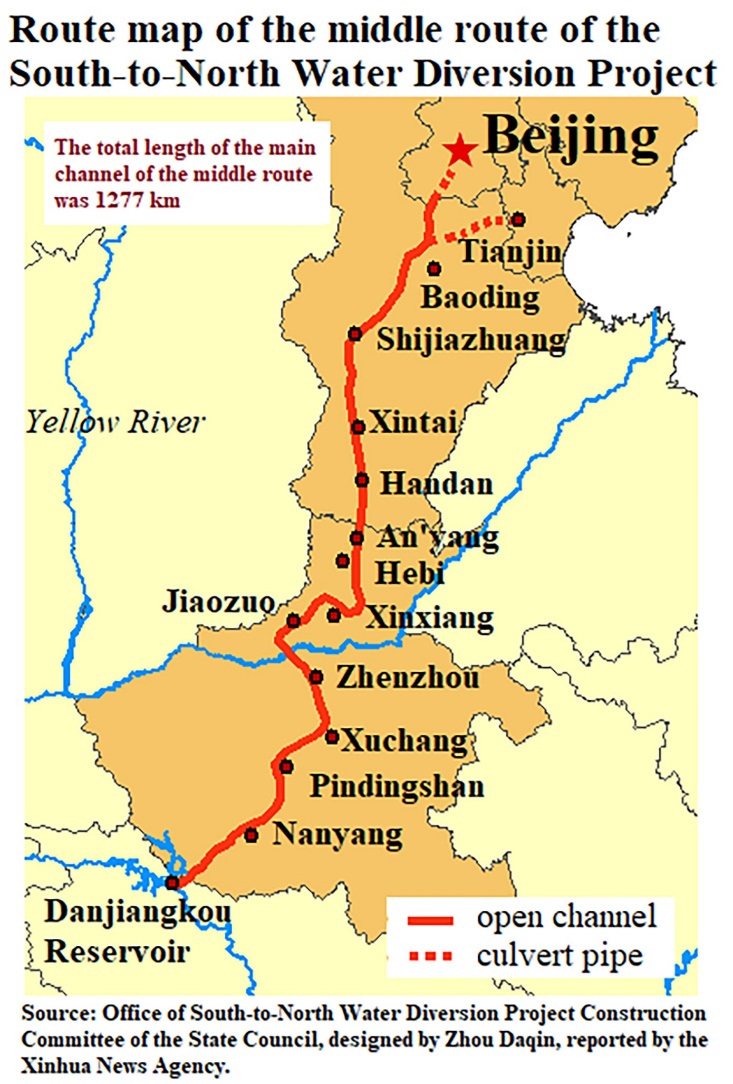


Fig. S1 Map of the Middle Route Project of the South-to-North Water Diversion Project which transports water from the Danjiangkou Reservoir (connected to the Yangtze River) to Henan and Hebei Provinces and to the Tianjin and Beijing municipalities

Table S1 Detailed information about the purpose, source and time period of different datasets

| Dataset | Purpose | Source | Time period |
| --- | --- | --- | --- |
| CDWS type and number | Highlight the vital importance of lakes and reservoirs evidenced by CDWSs number | Ministry of Ecology and Environment | Until 2020 |
| Population of cities at both prefecture and county levels | Estimate the serving population of each CDWS type in the city | https://www.hongheiku.com/ | 2019 |
| Monthly water quality data for the drinking water sources in Guangdong and Shandong Provinces | Compare the water quality among different drinking water sources | http://jcc.sdein.gov.cn/hjzl/  http://gdee.gd.gov.cn/xwfb4199/ | 2016-2020 |
| Monthly drinking water intake in Shanxi Province | Quantify the contributions of three types prefecture-level CDWSs to the drinking water supply | https://sthjt.shanxi.gov.cn/ | 2016-2020 |
| Yearly water supply and use | Quantify the increase of yearly water supply and use, relative contribution of surface water and groundwater | China water resources bulletin issued | 1997-2020 |
| Water diversion volume from Danjiangkou Reservoir | Underline cross-regional water transfer projects from lakes and reservoirs | Changjiang Water Resources Protection Institute | 2019 |

Table S2 Detailed information of water supply and use from 1997 to 2020 from China water resources bulletin issued by Ministry of Water Resources

|  | Water supply amount (10^8^ m^3^) | | | | Water supply percentage (%) | | | Water use amount (10^8^ m^3^) | | | | Water use percentage (%) | | |
| --- | --- | --- | --- | --- | --- | --- | --- | --- | --- | --- | --- | --- | --- | --- |
| Year | Surface water | Ground  water | Other | Total | Surface water | Ground water | Other | Domestic | Industrial | Agricultural and ecological | Total | Domestic | Industrial | Agricultural and ecological |
| 1997 | 4566 | 1029 | 28 | 5623 | 81.2 | 18.3 | 0.5 | 525 | 1121 | 3920 | 5566 | 9.4 | 20.2 | 70.4 |
| 1998 | 4420 | 1028 | 22 | 5470 | 80.8 | 18.8 | 0.4 | 544 | 1125 | 3766 | 5435 | 10.0 | 20.7 | 69.3 |
| 1999 | 4518 | 1072 | 22 | 5613 | 80.5 | 19.1 | 0.4 | 563 | 1159 | 3869 | 5591 | 10.1 | 20.7 | 69.2 |
| 2000 | 4440 | 1069 | 21 | 5531 | 80.3 | 19.3 | 0.4 | 575 | 1139 | 3784 | 5498 | 10.5 | 20.7 | 68.8 |
| 2001 | 4448 | 1097 | 22 | 5567 | 79.9 | 19.7 | 0.4 | 601 | 1141 | 3825 | 5567 | 10.8 | 20.5 | 68.7 |
| 2002 | 4403 | 1072 | 22 | 5497 | 80.1 | 19.5 | 0.4 | 616 | 1143 | 3738 | 5497 | 11.2 | 20.8 | 68.0 |
| 2003 | 4288 | 1016 | 16 | 5320 | 80.6 | 19.1 | 0.3 | 633 | 1176 | 3511 | 5320 | 11.9 | 22.1 | 66.0 |
| 2004 | 4505 | 1026 | 17 | 5548 | 81.2 | 18.5 | 0.3 | 649 | 1232 | 3667 | 5548 | 11.7 | 22.2 | 66.1 |
| 2005 | 4574 | 1036 | 23 | 5633 | 81.2 | 18.4 | 0.4 | 676 | 1284 | 3673 | 5633 | 12.0 | 22.8 | 65.2 |
| 2006 | 4706 | 1066 | 23 | 5795 | 81.2 | 18.4 | 0.4 | 695 | 1344 | 3755 | 5795 | 12.0 | 23.2 | 64.8 |
| 2007 | 4725 | 1071 | 23 | 5819 | 81.2 | 18.4 | 0.4 | 710 | 1402 | 3707 | 5819 | 12.2 | 24.1 | 63.7 |
| 2008 | 4799 | 1082 | 30 | 5910 | 81.2 | 18.3 | 0.5 | 727 | 1401 | 3782 | 5910 | 12.3 | 23.7 | 64.0 |
| 2009 | 4838 | 1098 | 30 | 5965 | 81.1 | 18.4 | 0.5 | 752 | 1390 | 3824 | 5965 | 12.6 | 23.3 | 64.1 |
| 2010 | 4884 | 1108 | 30 | 6022 | 81.1 | 18.4 | 0.5 | 765 | 1445 | 3812 | 6022 | 12.7 | 24.0 | 63.3 |
| 2011 | 4953 | 1112 | 43 | 6107 | 81.1 | 18.2 | 0.7 | 790 | 1462 | 3855 | 6107 | 12.9 | 23.9 | 63.2 |
| 2012 | 4954 | 1134 | 43 | 6131 | 80.8 | 18.5 | 0.7 | 742 | 1380 | 4010 | 6131 | 12.1 | 22.5 | 65.4 |
| 2013 | 5009 | 1125 | 49 | 6183 | 81.0 | 18.2 | 0.8 | 748 | 1410 | 4025 | 6183 | 12.1 | 22.8 | 65.1 |
| 2014 | 4925 | 1115 | 55 | 6095 | 80.8 | 18.3 | 0.9 | 768 | 1353 | 3974 | 6095 | 12.6 | 22.2 | 65.2 |
| 2015 | 4968 | 1068 | 67 | 6103 | 81.4 | 17.5 | 1.1 | 793 | 1337 | 3973 | 6103 | 13.0 | 21.9 | 65.1 |
| 2016 | 4911 | 1057 | 72 | 6040 | 81.3 | 17.5 | 1.2 | 822 | 1308 | 3911 | 6040 | 13.6 | 21.6 | 64.8 |
| 2017 | 4944 | 1015 | 85 | 6043 | 81.8 | 16.8 | 1.4 | 838 | 1277 | 3928 | 6043 | 13.9 | 21.1 | 65.0 |
| 2018 | 4951 | 975 | 90 | 6016 | 82.3 | 16.2 | 1.5 | 860 | 1262 | 3894 | 6016 | 14.3 | 21.0 | 64.7 |
| 2019 | 4983 | 934 | 104 | 6021 | 82.8 | 15.5 | 1.7 | 872 | 1218 | 3932 | 6021 | 14.5 | 20.2 | 65.3 |
| 2020 | 4792 | 893 | 128 | 5813 | 82.4 | 15.4 | 2.2 | 863 | 1031 | 3919 | 5813 | 14.8 | 17.7 | 67.4 |

Table S3 10 major drinking water pollution incidents occurring in China in the past 20 years

| Drinking water pollution incident | Effect and harm | Reason | Year | Drinking water source type |
| --- | --- | --- | --- | --- |
| Sichuan Tuojiang River water pollution accident | Million population in Jianyang, Zizhong and Neijiang were forced to stop drinking water | High concentration industrial wastewater polluted water source Tuojiang River | 2004 | Rivers type |
| Water pollution incident in Puyang, Henan Province | 0.4 million population in Puyang was affected for 4 months | Wastewater from fertilizer and paper mills polluted water intake of the Yellow River | 2004 | Rivers type |
| Water pollution incident of Qingyi River | 0.4 million population in Leshan was affected |  | 2004 | Rivers type |
| Songhua River pollution accident | Drinking water system serving Harbin’s 4 million population and groundwater supplies along the river’s edge were taken offline for 4 days | About 100 tons of benzene (benzene, nitrobenzene, etc.) polluted Songhua River | 2005 | Rivers type |
| Arsenic pollution in Yueyang, Hunan Province | 0.08 million population in Yueyang was affected | Industrial wastewater from three chemical plants polluted Xinqiang River | 2006 | Rivers type |
| Pollution accident of Luzhou power plant in Sichuan Province | Drinking water in Luzhou was affected | The leakage of diesel oil resulted in the Yangtze River water pollution | 2006 | Rivers type |
| Taihu water crisis event | 200 million population in Wuxi was affected | Toxin-producing cyanobacterial bloom polluted drinking water source in Lake Taihu | 2007 | Lakes and reservoirs type |
| Arsenic pollution in Lake Yangzonghai, Yunnan Province | Drinking water in Lake Yangzonghai was affected | Yunnan Chengjiang Jinye industry and Trade Co., Ltd. discharges a large amount of arsenic containing wastewater | 2008 | Lakes and reservoirs type |
| Water pollution incident in Yancheng, Jiangsu Province | 0.2 million population in Yancheng was affected | Yancheng BiaoXin chemical plant secretly discharged 30 tons of chemical wastewater, which eventually polluted the water source | 2009 | Rivers type |
| Cadmium pollution in Longjiang, Guangxi | Drinking water in Liuzhou in Longjiang was affected | Guangxi Jinhe Mining Co., Ltd. and Jincheng jianghongquan lithopone plant have long-term illegal discharge, resulting in large-scale water pollution | 2012 | Rivers type |

Table S4 Water diversion volume from Danjiangkou Reservoir to different prefecture-level cities along the Middle Route Project of the South-to-North Water Diversion Project (SNWD) in 2019

| Prefecture-level cities | Total water diversion volume (10^4^ m^3^) | Ecological water diversion volume (10^4^ m^3^) | Drinking water diversion volume (10^4^ m^3^) | Number of outlets | Number of sluice gates |
| --- | --- | --- | --- | --- | --- |
| Anyang | 12110 | 2018 | 10092 | 3 | 2 |
| Baoding | 114426 | 60660 | 53767 | 13 | 6 |
| Beijing | 68407 | 0 | 68407 | 1 | 0 |
| Handan | 44145 | 21920 | 22225 | 5 | 2 |
| Hebi | 17577 | 3205 | 14372 | 3 | 1 |
| Jiaozuo | 8511 | 652 | 7860 | 4 | 1 |
| Langfang | 3007 | 0 | 3007 | 2 | 0 |
| Nanyang | 105429 | 28355 | 77074 | 8 | 7 |
| Pingdingshan | 41051 | 4956 | 36095 | 6 | 5 |
| Shijiazhuang | 143947 | 54132 | 89815 | 10 | 4 |
| Tianjin | 129061 | 7204 | 121857 | 1 | 0 |
| Xinxiang | 18071 | 5396 | 12675 | 4 | 3 |
| Xingtai | 59582 | 36507 | 23075 | 5 | 5 |
| Xuchang | 25752 | 3622 | 22131 | 4 | 1 |
| Zhengzhou | 71130 | 11702 | 59427 | 7 | 5 |
| **Total** | **862206** | **240328** | **621878** | **76** | **42** |

Table S5 In operation and to be operated important cross-regional water transfer projects from lakes and reservoirs to meet safe and clean drinking water in China

| Number |  | Water sources | Water diversion amount (10^8^ m^3^/year) | Beneficiary regions | Beneficiary population (million) |
| --- | --- | --- | --- | --- | --- |
| 1 | East Route Project of SNWD | Lake Hongzehu, and Lake Luomahu | 17 | Zaozhuang, Jining, Tai’an, Dezhou, Liaocheng, Jinan, Heze, Laifu, Linyi, Zibo prefecture-level regions in Shandong province, Cangzhou and Hengshui prefecture-level regions in Hebei province, Tianjin municipality | 58 |
| 2 | Middle Route Project of SNWD | Danjiangkou Reservoir | 95 | Nanyang, Pingdingshan, Xuchang, Zhengzhou, Jiaozuo, Xinxiang, Hebi, Anyang prefecture-level regions in Henan province, Handan, Xingtai, Shijiazhuang, and Baoding prefecture-level regions in Hebei province, Beijing and Tianjin municipalities | 69 |
| 3 | Water Diversion Project from Hanjiang River to Weihe River | Huangjinxia Reservoir, Sanhekou Reservoir | 15 | Xi’an, Xianyang, Baoji, and Weinan prefecture-level regions in Shaanxi province | 20 |
| 4 | Lake Qiandao Water Distribution Project | Lake Qiandaohu | 12.1 | Hangzhou and Jiaxing prefecture-level regions in Zhejiang province | 10 |
| 5 | River Taohe Water Diversion Project | Jiudianxia Reservoir | 5.5 | Lanzhou, Dingxi, and Baiyin prefecture-level regions in Gansu province | 3 |
| 6 | Water Diversion Project from Yellow River to Shanxi Province | Wanjiazai Reservoir | 12 | Taiyuan, Suozhou, Datong prefecture-level regions in Shanxi province | / |
| 7 | Water diversion project from Datong to Huanghe | Shitouxia Reservoir and Heiquan Reservoir | 7.5 | Xining, Haibei, and Haidong prefecture-level regions in Qinghai province | 3 |
| 8 | Water diversion project from Lake Songhuahu | Lake Songhuahu | 7.3 | Changchun, Siping, and Liaoyuan prefecture-level regions in Jilin province | 10.6 |
